# Association between Ventilation Disorder and Masticatory Dysfunction in the Korean Adult Population: A Cross-Sectional Study Using Data from the 2019 Korea National Health and Nutrition Examination Survey

**DOI:** 10.3390/medicina60111779

**Published:** 2024-10-31

**Authors:** So-Yeong Kim, Sun-A Lim

**Affiliations:** 1 Department of Preventive Medicine, College of Medicine, Chosun University, Gwangju 61452, Republic of Korea; 2 Department of Dental Hygiene, Songwon University, Gwangju 61756, Republic of Korea

**Keywords:** chronic obstructive pulmonary disease, KNHANES, oral disease

## Abstract

*Background and Objectives*: Respiratory and periodontal diseases are among the most common diseases affecting humans worldwide. Periodontal disease is a risk factor for aspiration pneumonia and other respiratory diseases. However, the evidence supporting the link between respiratory and periodontal disease is inconclusive and insufficient. Therefore, this study aimed to investigate the relationship between oral disease symptoms and respiratory diseases. *Materials and Methods*: Adults who underwent oral examinations and lung disease tests were recruited from the 2019 Korea National Health and Nutrition Examination Survey (KNHANES). A total of 14,956 individuals participated in the 2019 KNHANES. Among the 6846 participants who underwent oral examinations, 1320 children and adolescents were excluded. Additionally, of the 4119 adults aged ≥40 years who underwent dual lung function tests, those who did not respond to key independent confounding variables were excluded. The final sample consisted of 2988 adults. *Results*: Complex sample multivariate logistic regression analysis revealed that the risk of restrictive ventilation disorder was lower in individuals without chewing discomfort compared to individuals with chewing discomfort (odds ratio, 0.68; 95% confidence interval, 0.479–0.996), and the difference was statistically significant. Only one significant difference was observed between the two groups. *Conclusions*: Chewing discomfort is a major periodontal health-related factor associated with chronic obstructive pulmonary disease (COPD). Although the exact mechanism underlying the relationship between masticatory discomfort and COPD remains unclear, masticatory discomfort is an early symptom of oral diseases. The findings from this study provide additional basic data for the prevention of oral diseases and COPD in Korea.

## 1. Introduction

Chronic obstructive pulmonary disease (COPD) is a heterogeneous lung disease characterized by dyspnea, cough, and phlegm owing to persistent abnormalities in the airways and alveoli, often resulting in progressive airflow limitation [1]. COPD is primarily caused by long-term inhalation exposure to pollutants such as cigarette smoke. According to the World Health Organization, COPD is one of the top 10 causes of death worldwide [2]. Inflammation due to COPD occurs not only in the lungs but also throughout the body, and COPD is associated with systemic comorbidities such as osteoporosis, diabetes, and cardiovascular disease [3]. The global prevalence of COPD in adults aged >40 years is 9–10%, and the annual number of deaths worldwide is estimated to be three million [4]. Moreover, patients with frequent or severe exacerbations are at increased risk of further exacerbations, resulting in a significant healthcare burden associated with COPD [5]. 

Respiratory diseases and periodontitis are among the most common diseases affecting humans worldwide. Periodontitis affects >50% of the adult population, and 11% of adults have severe periodontitis, making it the sixth most common disease in humans [6]. Additionally, pneumonia, a common infectious disease of the lung parenchyma, is more prevalent among older adults. The oral microbiota plays an important role in the natural history of certain forms of pneumonia [7], and periodontitis is a risk factor for aspiration pneumonia and other respiratory infections [8]. Patients with severe periodontitis have a high risk of developing pneumonia because of aspiration of pathogenic periodontal bacteria into the airways, and lung damage due to respiratory infections can increase the risk of COPD [9].

The inflammatory response is a possible link between impaired lung function and periodontal disease [10]. Recent studies have shown that periodontitis is a risk factor for chronic diseases related to systemic inflammation, such as diabetes, cardiovascular disease, and COPD [11]. Additionally, periodontal disease and pulmonary dysfunction share the same risk factors, such as age, obesity, smoking, socioeconomic status, and living conditions [12]. Therefore, we hypothesized that oral diseases are correlated with pulmonary dysfunction.

However, the evidence supporting the association between respiratory and periodontal disease is inconclusive and insufficient. Moreover, the evidence regarding the effect of periodontal treatment on the onset and progression of respiratory disease in patients with periodontal disease is limited [8,13,14], and studies focusing on the general Korean population are scarce. Therefore, this study aimed to determine the correlation between periodontal disease and COPD.

## 2. Materials and Methods

This manuscript has been prepared in accordance with the Strengthening the Reporting of Observational Studies in Epidemiology (STROBE) guidelines [15].

We analyzed data from the 2019 Korea National Health and Nutrition Examination Survey (KNHANES). The 2019 KNHANES is a national cross-sectional survey of people’s overall health status, chronic diseases, food and nutritional intake, and oral health status, with reliable data representing the national health status of Korea. A stratified cluster sample from the National Health and Nutrition Survey was used to select a nationally representative sample. The 2019 KNHANES included health surveys and health checkups through mobile checkup centers to collect participants’ overall and oral health statuses. A total of 30,490 individuals aged ≥1 year were selected for participation in the eighth KNHANES, of which 22,559 (74.0%) responded. The KNHANES protocol was approved by the Korea Disease Control and Prevention Agency Review Committee (2018-01-03-C-A), and written informed consent was obtained from each participant before conducting the surveys and tests. Oral examinations were jointly conducted by a dentist affiliated with the Korea Disease Control and Prevention Agency and a public health dentist.

A total of 14,956 individuals from the 2019 KNHANES were enrolled in this study. Among the 6846 participants who underwent oral examinations, 1320 children and adolescents were excluded. Additionally, of the 4119 adults aged ≥40 years who underwent dual lung function tests, those who did not respond to key independent confounding variables were excluded. The final sample consisted of 2988 adults.

Pulmonary function tests included forced vital capacity (FVC) and forced expiratory volume in 1 s (FEV1) assessment. Obstructive disorder, restrictive disorder, and normal lung function were defined as FEV1/FVC < 0.70, FEV1/FVC ≥ 0.70, and FVC < 80%, and FEV1/FVC ≥ 0.70 and FVC ≥ 80%, respectively [16,17].

The following factors related to the participants’ oral health were evaluated: history of toothache in the past year (yes or no), chewing discomfort (yes or no), oral examination within the past year (yes or no), visit to a dental hospital or clinic within the past year (yes or no), unsatisfactory dental care experience (yes, no, or no dental treatment required), presence of permanent tooth caries (yes or no), and history of permanent tooth caries (yes or no).

Regarding participants’ systemic health, a doctor’s diagnosis of one or more systemic diseases during their lifetime was evaluated. Hypertension, dyslipidemia, myocardial infarction, osteoarthritis, pulmonary tuberculosis, asthma, diabetes, and sinusitis were identified as systemic diseases. Considering that the dependent variable was lung disease, participants’ current smoking status was determined.

The confounding factors included sex (male or female), age (40–64 years), monthly household income (high, middle, or low), education level (elementary school or less, middle school, high school, college, or higher), and private health insurance subscription (yes or no).

The collected data were analyzed using SPSS 27.0 (IBM Corp., Armonk, NY, USA). All statistical analyses were performed using a complex sampling method. The Rao–Scott chi-square test was used to analyze confounding factors for the correlation between periodontal disease and COPD. Multinomial logistic regression analysis was used to determine the relationship between COPD and periodontal disease after excluding significant confounding variables from the Rao–Scott chi-square test. The significance level for all statistical analyses was set at *p* < 0.05. All analyses were performed using IBM SPSS 26.0 software (IBM Co., Armonk, NY, USA).

## 3. Results

### 3.1. Pulmonary Function According to Participants’ General Characteristics 

Lung function was normal in 74.5% of the participants, whereas restrictive and obstructive ventilatory disorders were observed in 13.5% and 12.0% of the participants. Normal lung function, restrictive ventilatory disorder, and obstructive ventilatory disorder were observed in 69.5%, 16.9%, and 17.3% of men; 82.8%, 10.2%, and 7.0% of women (*p* < 0.001); 80.9%, 11.6%, and 7.5% of those aged 40–64 years; and 53.6%, 19.8%, and 26.7% of those aged ≥65 years, respectively (*p* < 0.001). Statistically significant differences were observed in the household income, education level, and private health insurance subscriptions (*p* < 0.001) (Table 1).

### 3.2. Pulmonary Function According to Systemic Disease 

Lung function disorders, according to the diagnosis of systemic diseases by a physician, are shown in Table 2. Among patients with restrictive ventilation disorders, 10.5% of patients were not diagnosed with hypertension, whereas 21.5% were diagnosed with hypertension (*p* < 0.001). Additionally, statistically significant differences were observed in the prevalence of dyslipidemia, myocardial infarction, osteoarthritis, pulmonary tuberculosis, asthma, and diabetes; current smoking status; restrictive chills disorder; and obstructive ventilation disorder.

### 3.3. COPD Prevalence According to Participants’ Oral Health Characteristics 

Lung function disorders according to the participants’ oral health characteristics are presented in Table 3. Among participants with and without a history of toothache in the past year, 13.2% and 14.3% had restrictive ventilatory disorder. Furthermore, 11.1% and 15.2% had obstructive ventilatory disorder, and the difference was statistically significant (*p* = 0.045). Additionally, restrictive and obstructive ventilatory disorders were observed in 13.3% and 10.7% of participants without chewing discomfort, respectively. In contrast, among participants with chewing discomfort, the prevalence was 14.0% and 17.2%, respectively, and the difference was statistically significant (*p* < 0.001).

However, no significant differences in pulmonary dysfunction according to the history of oral examinations within the past year, visit to a dental hospital or clinic within the past year, unsatisfactory dental care, presence of permanent tooth caries, or history of permanent tooth caries were observed.

### 3.4. Relationship between Lung Disease and Dental Pain

Table 4 presents the results of the complex sample multivariate logistic regression analysis after adjusting for significant variables in a simple analysis. The prevalence of restrictive ventilation disorder was significantly different between participants with and without chewing discomfort (adjusted odds ratio, 0.68; 95% confidence interval, 0.479–0.996).

## 4. Discussion

The findings in this study confirmed that the risk of obstructive ventilation disorders was low in participants without chewing discomfort. In other words, the more discomfort one experiences when chewing, the higher the risk of developing an obstructive ventilation disorder. Thus, the study hypothesis that oral diseases are correlated with pulmonary dysfunction was accepted.

General health is closely related to oral health. Therefore, the interest in understanding the relationship between oral diseases and systemic diseases is increasing. Several studies have investigated the relationship between periodontal disease and COPD. A cross-sectional study evaluating the association between oral hygiene and periodontal health and COPD exacerbation in China reported that COPD exacerbation increased in individuals with fewer remaining teeth [18]. These results are consistent with those of previous studies highlighting the potential relationship between periodontitis and respiratory function [19,20].

In this study, masticatory discomfort was a major periodontal health-related factor associated with COPD. Although the exact mechanism underlying the relationship between masticatory discomfort and COPD remains unclear, masticatory discomfort is an early symptom of oral diseases. A previous study suggested that oral bacteria aspirated together with respiratory pathogens may attach to the respiratory epithelium and cause lung disease [21]. Additionally, some studies have shown that improving oral hygiene and reducing the oral bacterial load can reduce the risk of respiratory diseases and lower respiratory infections [22].

We highlight the need for collaboration between pulmonologists and dentists for COPD management. Poor oral health is a major risk factor for COPD exacerbation and a potential independent, modifiable condition [23]. Thus, the presence of periodontal disease should alert the dentist to the possible coexistence of COPD and prompt referral for further evaluation [24]. Future studies should investigate whether patients with COPD can benefit from routine oral health evaluations and provide important insights into the pathophysiological link between periodontal disease and COPD [25].

This study had some limitations. First, as the symptoms related to oral diseases were surveyed, the data were vulnerable to response bias. Second, although this cross-sectional study confirmed the relationship between oral disease symptoms and COPD, a definite causal relationship could not be established. Third, it has the limitation of being a retrospective case-control study. Despite these limitations, the major advantage of this study is that it established the relationship between oral disease symptoms and COPD based on systemic disease diagnosis and sociodemographic characteristics as confounding factors. Additionally, variables related to systemic diseases that were not included in previous studies were considered as confounding factors. Furthermore, this study used extensive data from the 2019 KNHANES, which included a large number of Korean adults, to confirm the relationship between oral disease symptoms and COPD, and provides basic data supporting the management of oral disease for COPD prevention. 

## 5. Conclusions

The results of this study confirmed the significant association between oral disease symptoms and COPD in Korean adults. However, further research is required to establish a causal relationship. Additionally, a cohort study is needed to determine the relationship between COPD and common oral diseases, such as dental caries and periodontal disease, as well as oral disease symptoms.

## Figures and Tables

**Table 1:** Pulmonary function according to participants’ general characteristics.

Variable	Normal Lung Function	Restrictive Ventilation Disorder	Obstructive Ventilatory Disorder	*p*-Value
Total	74.5 (1.0)	13.5 (0.7)	12.0 (0.7)	
Sex				<0.001
Male	65.9 (1.7)	16.9 (1.2)	17.3 (1.2)	
Female	82.8 (1.1)	10.2 (0.9)	7.0 (0.8)	
Age				<0.001
40–64	80.9 (1.0)	11.6 (0.8)	7.5 (0.7)	
≥65	53.6 (2.1)	19.8 (1.6)	26.7 (2.0)	
Household income				<0.001
Low	66.4 (1.9)	16.0 (1.3)	17.7 (1.5)	
Normal	75.5 (2.1)	12.8 (1.5)	11.6 (1.5)	
High	78.8 (1.4)	12.2 (1.1)	8.9 (0.9)	
Education level				<0.001
Elementary school	62.4 (2.4)	18.0 (1.8)	19.6 (2.1)	
Middle school	67.9 (3.5)	17.0 (2.5)	15.1 (2.4)	
High school	76.4 (1.8)	11.6 (1.2)	12.0 (1.3)	
≥College	81.0 (1.5)	1.2 (1.2)	6.9 (1.0)	
Private medical insurance				<0.001
Yes	77.4 (1.0)	12.8 (0.8)	9.8 (0.7)	
No	58.5 (2.5)	17.2 (1.9)	24.2 (2.2)	

**Table 2:** Pulmonary function according to systemic disease.

Variable	Normal Lung Function	Restrictive Ventilation Disorder	Obstructive Ventilatory Disorder	*p*-Value
Hypertension				<0.001
No	80.1 (1.0)	10.5 (0.7)	19.8 (9.4)	
Yes	59.5 (1.9)	21.5 (1.5)	18.9 (1.6)	
Dyslipidemia				<0.001
No	77.4 (1.0)	11.9 (0.7)	10.6 (0.8)	
Yes	65.1 (2.1)	18.4 (1.8)	16.5 (1.5)	
Myocardial infarction				<0.001
No	75.6 (1.0)	12.9 (0.8)	11.5 (0.7)	
Yes	50.3 (5.7)	33.1 (6.3)	16.5 (5.4)	
Osteoarthritis				0.007
No	75.9 (1.1)	13.0 (0.7)	11.1 (0.8)	
Yes	67.5 (2.7)	16.9 (2.2)	15.6 (2.1)	
Tuberculosis				<0.001
No	75.7 (1.0)	13.1 (0.7)	11.2 (0.7)	
Yes	55.1 (5.8)	21.1 (4.4)	23.8 (4.6)	
Asthma				<0.001
No	75.6 (1.0)	13.2 (0.7)	11.2 (0.7)	
Yes	51.4 (6.9)	20.6 (5.0)	28.0 (5.6)	
Diabetes				<0.001
No	76.3 (1.1)	12.2 (0.7)	11.5 (0.8)	
Yes	58.5 (3.2)	24.8 (3.0)	16.7 (2.2)	
Sinusitis				0.090
No	75.4 (1.0)	13.4 (0.7)	11.2 (0.7)	
Yes	68.1 (4.9)	13.7 (2.8)	18.1 (4.1)	
Smoking				<0.001
No	75.9 (1.1)	13.5 (0.8)	10.6 (0.8)	
Yes	68.1 (2.5)	13.1 (1.9)	18.8 (2.1)	

**Table 3:** Chronic obstructive pulmonary disease prevalence according to participants’ oral health characteristics.

Variable	Normal Lung Function	Restrictive Ventilation Disorder	Obstructive Ventilatory Disorder	*p*-Value
Tooth pain				0.045
No	75.7 (1.1)	13.2 (0.9)	11.1 (0.8)	
Yes	70.5 (2.1)	14.3 (1.6)	15.2 (1.7)	
Chewing discomfort				<0.001
No	75.9 (1.2)	13.3 (0.8)	10.7 (0.8)	
Yes	68.9 (2.1)	14.0 (1.5)	17.2 (1.8)	
Oral examination				0.480
No	73.5 (1.3)	14.0 (1.0)	12.6 (1.0)	
Yes	75.9 (1.7)	12.7 (1.1)	11.4 (1.1)	
Visit to dental facility				0.794
No	75.2 (1.5)	12.8 (1.3)	12.0 (1.1)	
Yes	74.1 (1.4)	138.(0.9)	12.1 (0.9)	
Unmet dental care needs				0.281
Yes	76.2 (1.8)	13.2 (1.3)	10.6 (1.2)	
No	72.8 (1.4)	14.1 (0.9)	13.1 (1.0)	
No treatment	77.0 (2.0)	12.0 (1.6)	11.0 (1.4)	
Current dental caries				0.986
No	74.0 (4.6)	11.3 (3.1)	14.7 (3.7)	
Yes	74.5 (1.1)	13.6 (0.8)	11.9 (0.7)	
Previous dental caries experience				0.605
No	74.0 (4.6)	11.3 (3.1)	14.7 (3.7)	
Yes	74.5 (1.1)	13.6 (0.8)	11.9 (0.7)	

**Table 4:** Relationship between lung disease disorder and dental pain.

	Restrictive Ventilation Disorder aOR (95% CI)	Obstructive Ventilatory Disorder aOR (95% CI)
Tooth pain		
Yes	1.000	1.000
No	0.889 (0.628–1.260)	1.298 (0.918–1.834)
Chewing discomfort		
Yes	1.000	1.000
No	0.680 (0.479–0.966)	0.943 (0.668–1.332)

aOR, adjusted odds ratio; CI, confidence interval; Adjusted: sex, age, household income, education level, private medical insurance, hypertension, dyslipidemia, myocardial infarction, osteoarthritis, tuberculosis, asthma, diabetes, smoking.

## Data Availability

The data are available on the official KNHANES website (https://knhanes.kdca.go.kr/knhanes/sub03/sub03_02_05.do; accessed on 4 April 2024).
